# Terahertz graphene-based multi-functional anisotropic metamaterial and its equivalent circuit model

**DOI:** 10.1038/s41598-023-30605-z

**Published:** 2023-03-01

**Authors:** Somayyeh Asgari, Tapio Fabritius

**Affiliations:** Optoelectronics and Measurement Techniques Research Unit, Faculty of Information Technology and Electrical Engineering, University of Oulu, Oulu, Finland

**Keywords:** Electrical and electronic engineering, Optics and photonics

## Abstract

In this paper, a graphene-based multi-functional anisotropic metamaterial composed of two finite parallel graphene ribbons in each unit cell is designed and proposed in the 0.1–5.5 terahertz (THz) region. Simulations are performed by the finite element method (FEM) in the frequency-domain solver of CST Software. An equivalent circuit modeling (ECM) as a simplified approach has been provided by a MATLAB code to model the performance of the metamaterial. The metastructure is polarization-sensitive because of the geometric non-symmetry. The absorption/reflection spectrum of the metamaterial is dynamically tunable by changing the Fermi energy level of the graphene. The introduced metamaterial can act as a THz switch and inverter at 1.23 and 4.21 THz. It acts as an ON state when the incident electric field is in the x-direction and acts as an OFF state when the incident electric field is in the y-direction. It can also act as a bi-functional mirror: a triple-band mirror for the incident electric field in the x-direction and an ultra-broadband mirror for the incident electric field in the y-direction. The proposed metamaterial has a maximum absorption of 100%, maximum linear dichroism (LD) of 100%, and a maximum switching extinction ratio of 33.01 dB. The metamaterial and its applications could be used as a potential platform in future THz devices and systems.

## Introduction

Chiral metamaterials do not superimpose on their mirror image at any degree of rotation and can exhibit responses such as circular dichroism (CD) and linear dichroism (LD). CD may be observed from anisotropic metamaterials as well. However, since the origin of CD of chiral and anisotropic metamaterials is not the same, we are referring to CD_chi_ when the CD is caused by chirality and CD_ani_ while the corresponding response is caused by anisotropy. CD_chi_ ≠ 0 shows that the metamaterial is chiral. If CD_chi_ = 0, the metamaterial is not chiral in most cases. CD_ani_ ≠ 0 proves that the metamaterial is anisotropic. Anisotropy is included in both CD_chi_ and CD_ani_ definitions so we cannot conclude anything about the metamaterial anisotropy if only CD_ani_ = 0. A metamaterial can be anisotropic (showing non-equal responses to the incident waves in some different directions) while CD_ani_ = 0 but LD ≠ 0. Chirality can arise from chiral metamaterials or chiral molecules as sub-structures when anisotropy is introduced through metamaterials with anisotropic geometries, anisotropic material in the metamaterial structure, or applying a magnetic field. Metamaterials with anisotropic geometries do not superimpose on their mirror image at some degrees of rotation and can thus produce LD and/or CD_ani_^1,2^.

Graphene, a 2D layer of graphite, has excellent features which make it a promising candidate for optical devices and systems. Graphene-based chiral and anisotropic metamaterials have been proposed, analyzed, and investigated recently. They produce tunable CD (CD = CD_chi_ + CD_ani_) and/or LD responses up to 99%^3–12^. The design and investigation of tunable graphene-based metamaterials for switching, inverting, modulating, sensing, and so on applications is a worthy and uninvestigated field of research.

The recently reported Graphene-based chiral and anisotropic metamaterials^3–12^ have one to four bands. Except for Ref.^8^ which is a dual-functional mirror containing two layers of graphene-based resonators (broadband and multiband mirrors), while the others are investigated and analyzed as a single purpose device like absorber. The tunability feature of these metamaterials widens their applicability making them the interesting possibility for future telecommunication technology or spectroscopic sensing for example. There is an urgent need for tunable multi-functional metamaterial in THz systems to minimize the size of the system greatly as the metamaterial can have more than two performances at the same time and extend their versatility. The multi-functional device can be switched into different applications which is very beneficial. Moreover, we could save material, cost, and time greatly. In these papers, the maximum dichroism responses reached 99%.

Graphene-based metamaterials containing two parallel graphene ribbons in each unit cell were proposed and designed as filtering applications in Refs.^13,14^. The metamaterial in Ref.^14^ contains ribbons that are infinite from one side and finite from another side. The metamaterials don’t contain metal layers beneath the structures to avoid transmission and they were analyzed from the transmission point of view. The considered frequency region for Ref.^13^ is 13–30 THz and for Ref.^14^ is 4–26 THz. The metamaterial in Ref.^13^ doesn’t contain any theoretical analysis and the structure in Ref.^14^ is analyzed based on coupled mode theory.

In our earlier papers^7,10^, we proposed single-function graphene-based multi-band metamaterial absorbers containing a single layer of graphene resonators in 0.5–4.5 and 1–5.5 THz, respectively. The maximum LD responses reached 94 and 99%, respectively. In our earlier paper^8^, we proposed dual-function (multiband and broadband) graphene-based metamirror containing two layers of graphene resonators in 0.3–4.5 THz with a maximum LD response of 96%. In this paper, we propose a multi-functional graphene-based anisotropic metamaterial composed of one layer of parallel graphene ribbon resonators in 0.1–5.5 THz. The metamaterial is designed to act as a triple-band and ultra-broadband mirrors, inverter, and switch. Compared to Ref.^8^, we need fewer resources and time to analyze and perform the simulations and ECM. The ECM and its procedure in this work differ from that reported in Refs.^7,8,10^ as the graphene resonators are modeled as an impedance circuit in the x-direction and an open circuit (OC) in the y-direction. In addition, the number of layers containing the metamaterial differs from that reported in Refs.^7,8,10^.

## Multi-functional anisotropic metamaterial and equivalent circuit model

**Figure 1:**
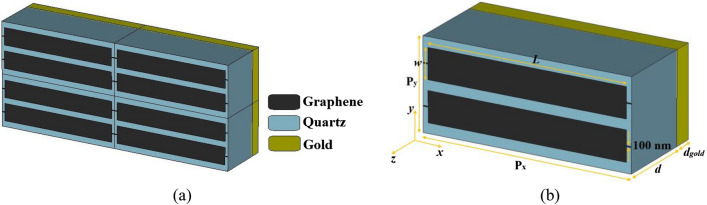
(**a**) Periodic and (**b**) unit cell views of the tunable graphene-based multi-functional anisotropic metamaterial composed of two parallel graphene ribbons in each unit cell. The substrate is made of Quartz and a metal gold layer is used beneath the metamaterial to avoid transmission.

Periodic and unit cell views of the designed graphene-based multi-functional THz anisotropic metamaterial comprised of supercell each cell containing two finite parallel ribbon resonators are given in Fig. 1. Thin graphene strips with a width of 100 nm are used to bias the graphene ribbon resonators^15,16^. The substrate is made of quartz with a refractive index of 1.96^17^. A gold metal layer with a conductivity of 4.56 × 10^7^ S/m^18^ is used beneath the metamaterial to avoid passing the electromagnetic waves from the other side of it. Simulations were done in the frequency domain solver of CST Microwave Studio by finite element method (FEM)^6–8,10^. Periodic boundary condition in the x- and y-directions, and absorbing boundary condition in the z-direction were used. The metamaterial was meshed by the tetrahedral mesh type. The device works as a tunable THz multi-functional metamaterial with four performances: switch, inverter, ultra-broadband mirror, and triple-band mirror. The dimensions and their optimized values are given in Table 1.

**Table 1:** The dimensions and their optimized values for the multi-functional anisotropic metamaterial of Fig. 1.

Parameter	Value (μm)	Parameter	Value (μm)	Parameter	Value
*L*	20	*w*	3	P_x_	21
P_y_	9	*d*	8	*d*_*gold*_	0.5

The metamaterial is optimized in CST by use of the genetic algorithm optimization technique^8,19^. The unit cell dimensions are assumed as *P*_*x*_ = 21 μm and *P*_*y*_ = 9 μm which are smaller than *λ*_*min*_ = 54.55 μm if *f*_*max*_ = 5.5 THz (maximum frequency of the simulated frequency range) to prevent the propagation of the high order Floquet modes^20–22^.

The total thickness of the metamaterial (graphene/quartz/gold layers) is ~ 8.5 μm (~ 0.15 × *λ*_*min*_) in the considered frequency range. So, the thickness of the metamaterial has been relatively thin.

The Fermi energy level of the graphene resonator layer *E*_*f*_ is assumed to be 1 eV for the graphene resonator layer. The relative permittivity of graphene is assumed by Refs.^7,8,10^:

1 $$ \varepsilon = 1 - j\frac{\sigma }{{\omega \varepsilon_{0} \Delta }}, $$

in which *σ*, *ω*, *ε*_0_, and Δ are the surface conductivity of graphene, angular frequency, permittivity of vacuum, and the thickness of graphene. Δ is assumed as 0.335 nm. *σ* contains the summation of the inter- and intra-band electron transition contributions based on the Kubo formula as follows^6,7,10,23–25^:

2a $$ \sigma = \sigma_{{{\text{inter}}}} \left( \omega \right) + \sigma_{{{\text{intra}}}} \left( \omega \right), $$

2b $$ \sigma_{{{\text{inter}}}} \left( \omega \right) = \frac{{e^{2} }}{4\hbar }\left[ {H\left( {\frac{\omega }{2}} \right) - \frac{4j\omega }{\pi }\int_{0}^{\infty } {\frac{{H\left( \xi \right) - H\left( {\frac{\omega }{2}} \right)}}{{\omega^{2} - 4\xi^{2} }}d\xi } } \right], $$

2c $$ \sigma_{{{\text{intra}}}} \left( \omega \right) = \frac{{2k_{B} e^{2} T}}{{\pi \hbar^{2} }}\ln \left[ {2\cosh \left( {\frac{{E_{f} }}{{2k_{B} T}}} \right)} \right]\frac{j}{{j\tau^{ - 1} - \omega }}, $$

2d $$ H\left( \xi \right) = \frac{{\sinh \left( {\frac{\hbar \xi }{{k_{B} T}}} \right)}}{{\cosh \left( {\frac{{E_{f} }}{{k_{B} T}}} \right) + \cosh \left( {\frac{\hbar \xi }{{k_{B} T}}} \right)}}, $$

where ℏ is the reduced Plank’s constant, *k*_*B*_ = 1.38 × 10^–23^ J/K is Boltzmann’s constant, *e* = 1.6 × 10^–19^ C is the electron charge, *T* is the temperature (300 K), and *ζ* is the integral variable. *τ* is the relaxation time^6,7,26^:

3 $$ \tau = \frac{{\mu E_{f} }}{{ev_{f}^{2} }}, $$

where *v*_*f*_ = 10^6^ m/s is the Fermi velocity and *µ* = 2 m^2^/(V s) is the carrier mobility of graphene. The propagation constant of the electromagnetic wave in a graphene-vacuum configuration is^6,7,27^:

4 $$ \beta = k_{0} \sqrt {1 - \left( {\frac{2}{{\eta_{0} \sigma }}} \right)^{2} } , $$

where *k*_0_ and *η*_0_ are the wave vector of the incident wave and the vacuum impedance.

The graphene Fermi energy level *E*_*f*_ could be controlled by the applied external bias voltage. The relation between *E*_*f*_ and the applied bias voltage can be expressed as^6,28^:

5 $$ \left| {E_{f} \left( V \right)} \right| = \hbar v_{f} \sqrt {\pi \left| {a_{0} \left( {V - V_{0} } \right)} \right|} , $$

where *V*_0_ is the offset voltage^6,28^ and

6 $$ a_{0} = \frac{{\varepsilon_{0} \varepsilon_{d} }}{ed}, $$

in which *a*_0_ is the capacitive model of the structure, *ε*_*d*_ is the dielectric permittivity, and *V* is the externally applied bias voltage to the graphene resonator layer.

**Figure 2:**
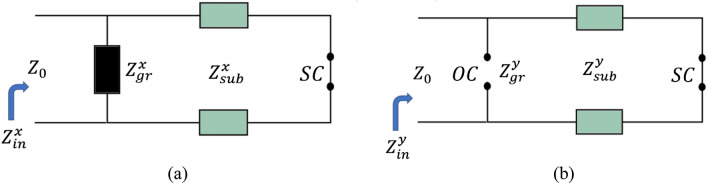
The equivalent circuit models (ECMs) of the proposed multi-functional metamaterial of Fig. 1 for the incident electric field (E field) in (**a**) x- and (**b**) y-directions. When the E field is parallel to the length of the resonators, the resonators are modeled as an impedance. When the E field is parallel to the width of the resonators, the resonators are modeled as an open circuit (OC). The metallic gold layer beneath the metamaterial is modeled by a short circuit (SC).

The multi-functional metamaterial in Fig. 1 is illuminated two times separately by the incident electric field E in x- and y- directions, respectively. When the metamaterial is illuminated by the E field in the x-direction (E field parallel to the length of the ribbons), the graphene resonator layer is modeled as an impedance $Z_{gr}^{x}$. When the metamaterial is excited by the E field in the y-direction (E field parallel to the width of the ribbons), the graphene resonator layer is modeled by an open circuit (OC). The gold metal layer is modeled as a short circuit (SC) in both states. The equivalent circuit models (ECMs) of the proposed multi-functional metamaterial with two illumination conditions are given in Fig. 2a,b.

The reflection coefficient $r^{x}$ is calculated in CST Software for the configuration containing the graphene resonator layer on the dielectric half-space with a thickness of 500 µm^7,8,10^. Then, the equivalent conductivity in the x-direction $\sigma_{gr}^{x}$ is calculated by the Fresnel equation^29^:

7 $$ \sigma_{gr}^{x} = \frac{{\sec \left( {\theta_{in} } \right) - \sqrt {\varepsilon_{{r_{sub} }} } \sec \left( {\theta_{out} } \right) - r^{x} \left( {\sec \left( {\theta_{in} } \right) + \sqrt {\varepsilon_{{r_{sub} }} } \sec \left( {\theta_{out} } \right)} \right)}}{{Z_{0} \left( {1 + r^{x} } \right)}}. $$

In which *θ*_*in*_, $\varepsilon_{{r_{sub} }}$, *θ*_*out*_, and *Z*_0_ are respectively the angle of the incident illuminated wave, the relative permittivity of the dielectric substrate (Quartz), the angle of the transmitted wave, and the vacuum impedance (377 Ω). The graphene resonator layer is modeled as an OC for the wave illumination in the y-direction. So:

8 $$ Z_{gr}^{y} = \infty , $$

9 $$ Y_{gr}^{y} = \frac{1}{{Z_{gr}^{y} }} = \frac{1}{\infty } = 0, $$

10 $$ \sigma_{gr}^{y} = Y_{gr}^{y} = 0. $$

The relation between *θ*_*in*_ and *θ*_*out*_ is:

11 $$ \sin \left( {\theta_{out} } \right) = \sqrt {\frac{1}{{\varepsilon_{{r_{sub} }} }}} \sin \left( {\theta_{in} } \right). $$

The transfer matrices of the graphene resonator layer in the x- and y-directions are as follows:

12 $$ \left[ {\varphi_{gr}^{x} } \right] = \left[ {\begin{array}{*{20}c} 1 & 0 \\ {\sigma_{gr}^{x} } & 1 \\ \end{array} } \right], $$

13 $$ \left[ {\varphi_{gr}^{y} } \right] = \left[ {\begin{array}{*{20}c} 1 & 0 \\ 0 & 1 \\ \end{array} } \right] = I. $$

The transfer matrix of the dielectric substrate in the x- or y-direction is:

14 $$ \left[ {\varphi_{sub}^{x/y} } \right] = \left[ {\begin{array}{*{20}l} {\cosh \left( {\theta_{sub} } \right)} & {Z_{sub}^{x/y} \sinh \left( {\theta_{sub} } \right)} \\ {\frac{1}{{Z_{sub}^{x/y} }}\sinh \left( {\theta_{sub} } \right)} & {\cosh \left( {\theta_{sub} } \right)} \\ \end{array} } \right], $$

in which *θ*_*sub*_ and $Z_{sub}^{x/y}$ are respectively the electrical length and the impedance of the dielectric substrate in the x- or y-direction. *θ*_*sub*_ is calculated by:

15 $$ \theta_{sub} = \frac{{jd\omega \sqrt {\varepsilon_{{r_{sub} }} } }}{c}, $$

in which *c* is the speed of the light. $Z_{sub}^{x}$ is:

16 $$ Z_{sub}^{x} = Z_{0} \sec \left( {\theta_{sub} } \right). $$

So:

17 $$ Z_{sub}^{x} = Z_{0} \sec \left( {\frac{{jd\omega \sqrt {\varepsilon_{{r_{sub} }} } }}{c}} \right). $$

$$Z_{sub}^{y}$$ is:

18 $$ Z_{sub}^{y} = Z_{0} \cos \left( {\theta_{sub} } \right). $$

So:

19 $$ Z_{sub}^{y} = Z_{0} \cos \left( {\frac{{jd\omega \sqrt {\varepsilon_{sub} } }}{c}} \right). $$

The total transfer matrix of the designed multi-functional metamaterial is:

20 $$ \left[ {\varphi_{tot}^{x/y} } \right] = \left[ {\varphi_{gr}^{x/y} } \right] \times \left[ {\varphi_{sub}^{x/y} } \right], $$

which is equal to:

21 $$ \left[ {\varphi_{tot}^{x/y} } \right] = \left[ {\begin{array}{*{20}c} {\varphi_{11}^{x/y} } & {\varphi_{12}^{x/y} } \\ {\varphi_{21}^{x/y} } & {\varphi_{22}^{x/y} } \\ \end{array} } \right]. $$

The matrix elements for the incident E field in the x-direction are:

22 $$ \varphi_{11}^{x} = \cosh \left( {\theta_{sub} } \right), $$

23 $$ \varphi_{12}^{x} = Z_{sub}^{x} \sinh \left( {\theta_{sub} } \right), $$

24 $$ \varphi_{21}^{x} = \sigma_{gr}^{x} \cosh \left( {\theta_{sub} } \right) + \frac{1}{{Z_{sub}^{x} }}\sinh \left( {\theta_{sub} } \right), $$

25 $$ \varphi_{22}^{x} = Z_{sub}^{x} \sigma_{gr}^{x} \sinh \left( {\theta_{sub} } \right) + \cosh \left( {\theta_{sub} } \right). $$

The matrix elements for the incident E field in the y-direction are:

26 $$ \varphi_{11}^{y} = \cosh \left( {\theta_{sub} } \right), $$

27 $$ \varphi_{12}^{y} = Z_{sub}^{y} \sinh \left( {\theta_{sub} } \right), $$

28 $$ \varphi_{21}^{y} = \frac{1}{{Z_{sub}^{y} }}\sinh \left( {\theta_{sub} } \right), $$

29 $$ \varphi_{22}^{y} = \cosh \left( {\theta_{sub} } \right). $$

The input impedance of the multi-functional metamaterial of Fig. 1 in the x- or y-direction is:

30 $$ Z_{in}^{x/y} = \frac{{\varphi_{12}^{x/y} }}{{\varphi_{22}^{x/y} }}, $$

by substituting Eqs. (23) and (25) in Eq. (30) for the x-direction, we have:

31 $$ Z_{in}^{x} = \frac{{Z_{sub}^{x} \sinh \left( {\theta_{sub} } \right)}}{{Z_{sub}^{x} \sigma_{gr}^{x} \sinh \left( {\theta_{sub} } \right) + \cosh \left( {\theta_{sub} } \right)}}, $$

by substituting Eqs. (27) and (29) in Eq. (30) for the y-direction, we have:

32 $$ Z_{in}^{y} = \frac{{Z_{sub}^{y} \sinh \left( {\theta_{sub} } \right)}}{{\cosh \left( {\theta_{sub} } \right)}}. $$

The scattering parameter in the x-direction is:

33 $$ S_{11}^{x} = \frac{{Z_{in}^{x} - Z_{0} \sec \left( {\theta_{in} } \right)}}{{Z_{in}^{x} + Z_{0} \sec \left( {\theta_{in} } \right)}}, $$

which is equal to:

34 $$ S_{11}^{x} = \frac{{Z_{sub}^{x} \sinh \left( {\theta_{sub} } \right) - Z_{0} \sec \left( {\theta_{in} } \right)\left[ {Z_{sub}^{x} \sigma_{gr}^{x} \sinh \left( {\theta_{sub} } \right) + \cosh \left( {\theta_{sub} } \right)} \right]}}{{Z_{sub}^{x} \sinh \left( {\theta_{sub} } \right) + Z_{0} \sec \left( {\theta_{in} } \right)\left[ {Z_{sub}^{x} \sigma_{gr}^{x} \sinh \left( {\theta_{sub} } \right) + \cosh \left( {\theta_{sub} } \right)} \right]}}. $$

The scattering parameter in the y-direction is:

35 $$ S_{11}^{y} = \frac{{Z_{in}^{y} - Z_{0} \cos \left( {\theta_{in} } \right)}}{{Z_{in}^{y} + Z_{0} \cos \left( {\theta_{in} } \right)}}, $$

which is equal to:

36 $$ S_{11}^{y} = \frac{{Z_{sub}^{y} \sinh \left( {\theta_{sub} } \right) - Z_{0} \cos \left( {\theta_{in} } \right)\cosh \left( {\theta_{sub} } \right)}}{{Z_{sub}^{y} \sinh \left( {\theta_{sub} } \right) + Z_{0} \cos \left( {\theta_{in} } \right)\cosh \left( {\theta_{sub} } \right)}}. $$

The reflection coefficients in x- or y-directions are:

37 $$ R^{x/y} = \left| {S_{11}^{x/y} } \right|^{2} . $$

The linear dichroism (LD) is calculated by:

38 $$ LD = A^{x} - A^{y} , $$

in which $A^{x}$ and $A^{y}$ are respectively the absorptions of the multi-functional metamaterial in the x- and y-directions.

The extinction ratio (ER) of the multi-functional metamaterial in the switching performance in dB is calculated by:

39 $$ ER = 10\log \left( {\frac{{A^{x} }}{{A^{y} }}} \right). $$

## Results and discussion

**Figure 3:**
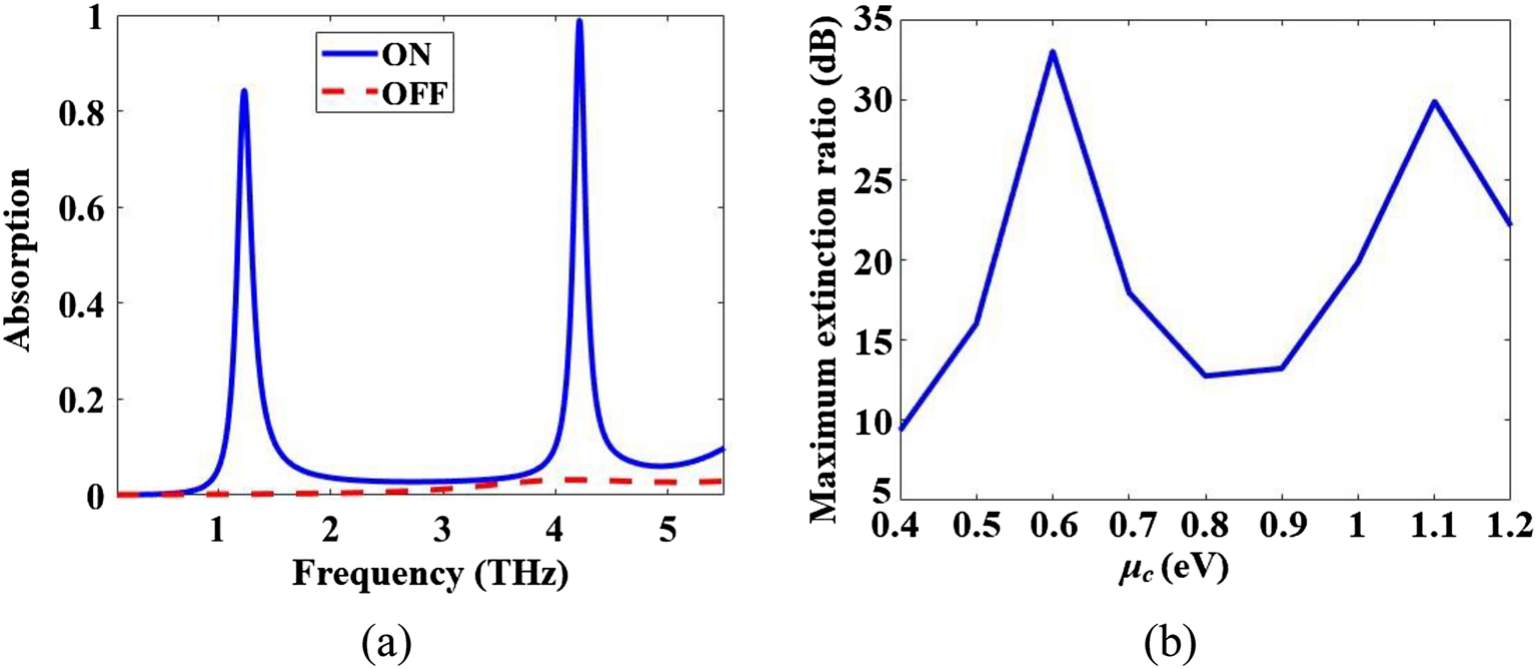
(**a**) Absorption spectra of the multi-functional metamaterial of Fig. 1 in switching performance for ON and OFF states. (**b**) The maximum extinction ratio in dB vs *µ*_*c*_ (eV) in switching performance of the metamaterial.

The absorption spectra of the multi-functional metamaterial of Fig. 1 in switching performance are depicted in Fig. 3a. By rotating the incident E-field from the x-direction to the y-direction, the metamaterial can switch from the “ON” state to the “OFF” state. The maximum extinction ratios (ERs) in dB by Eq. (39) vs *µ*_*c*_ (eV) are obtained for the switching performance of the multi-functional metamaterial in Fig. 1 and the results are given in Fig. 3b. The maximum obtained ER is 33.01 dB which occurs for *µ*_*c*_ = 0.6 eV.

**Figure 4:**
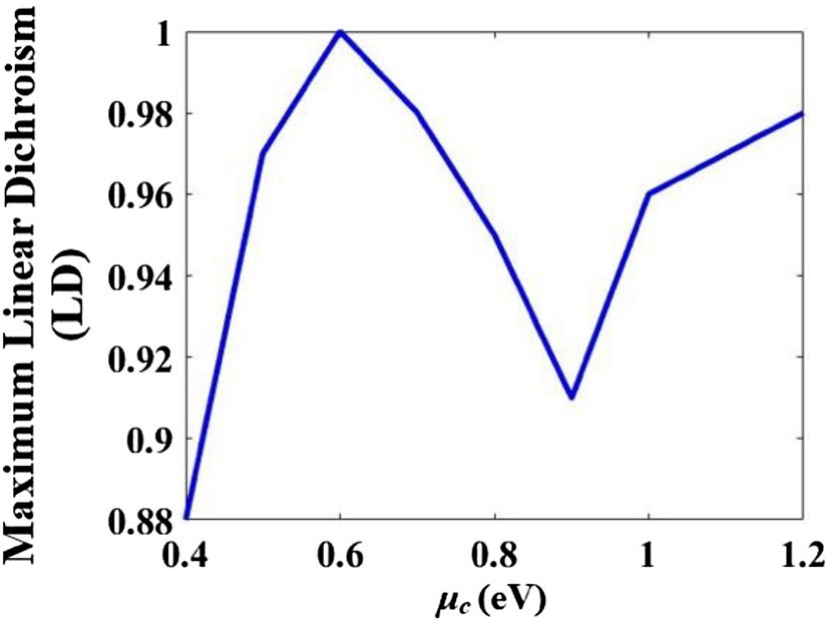
The maximum linear dichroism (LD) vs *µ*_*c*_ (eV) of the metamaterial.

The maximum linear dichroisms (LDs) vs *µ*_*c*_ (eV) are obtained for the anisotropic metamaterial by Eq. (38) and the results are given in Fig. 4. The maximum LD reaches 100% when *µ*_*c*_ = 0.6 eV. CD_ani_ = 0 (based on Eq. (10) in Ref.^2^).

**Figure 5:**
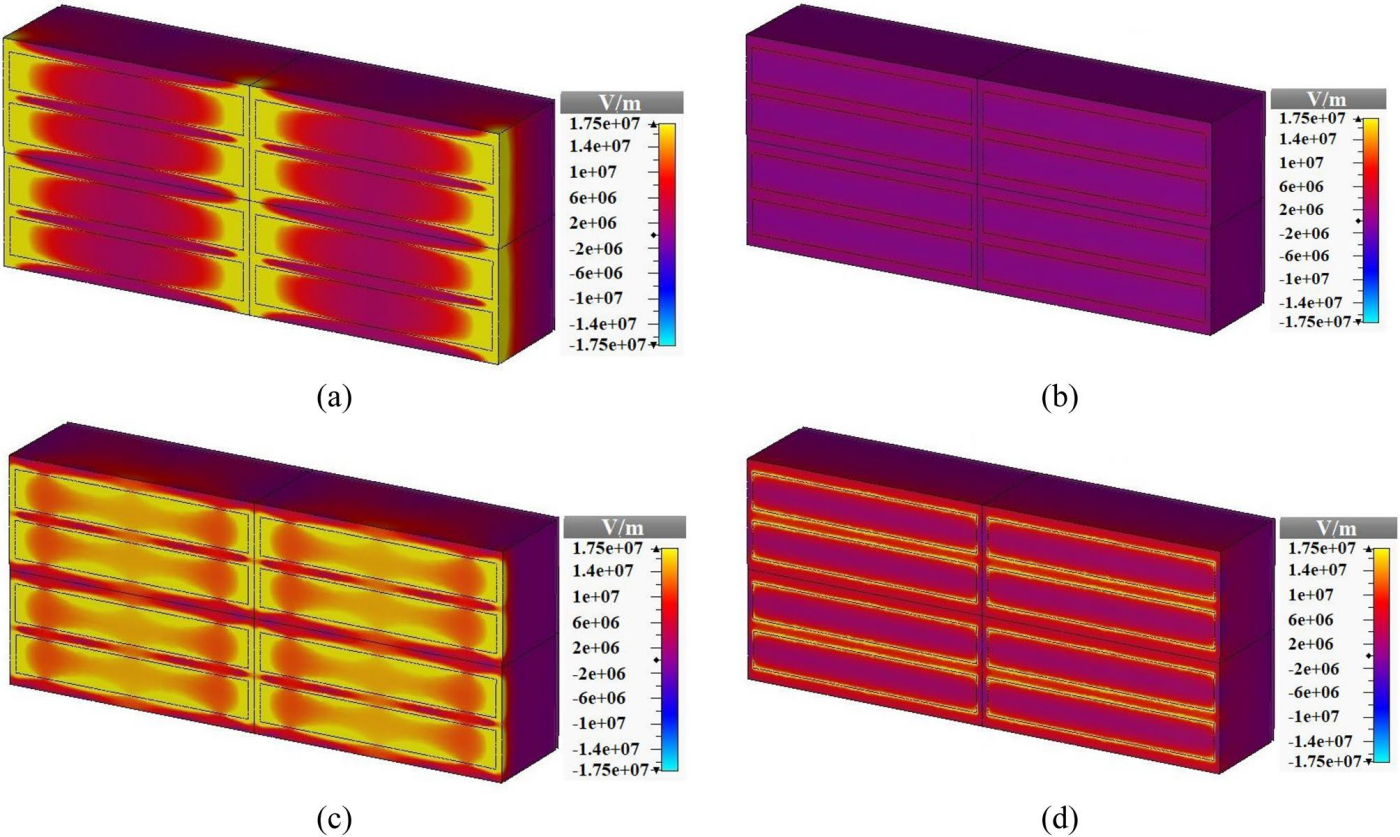
E field distributions of the metamaterial of Fig. 1 for the incident (**a**) E field in the x-direction at 1.23 THz, (**b**) E field in the y-direction at 1.23 THz, (**c**) E field in the x-direction at 4.21 THz, and (**d**) E field in the y-direction at 4.21 THz.

The E field distributions of the multi-functional metamaterial of Fig. 1 at 1.23 THz when the incident E field is in the x- and y-directions are respectively given in Fig. 5a,b. Also the E field distributions are given at 4.21 THz for the incident E field in the x- and y-directions in Fig. 5c,d, respectively. As it is clear, the distributions for the incident E field in the x- and y-directions at 1.23 (or 4.21) THz are not equal representing the validity of absorption spectra in both x- and y- directions. The metamaterial resonates for the incident E field in the x-direction at 1.23 and 4.21 THz which is shown in Fig. 5a,c. The metamaterial doesn’t resonate at all in the whole frequency range when the incident E field is in the y-direction. This is shown in Fig. 5b,d.

**Figure 6:**
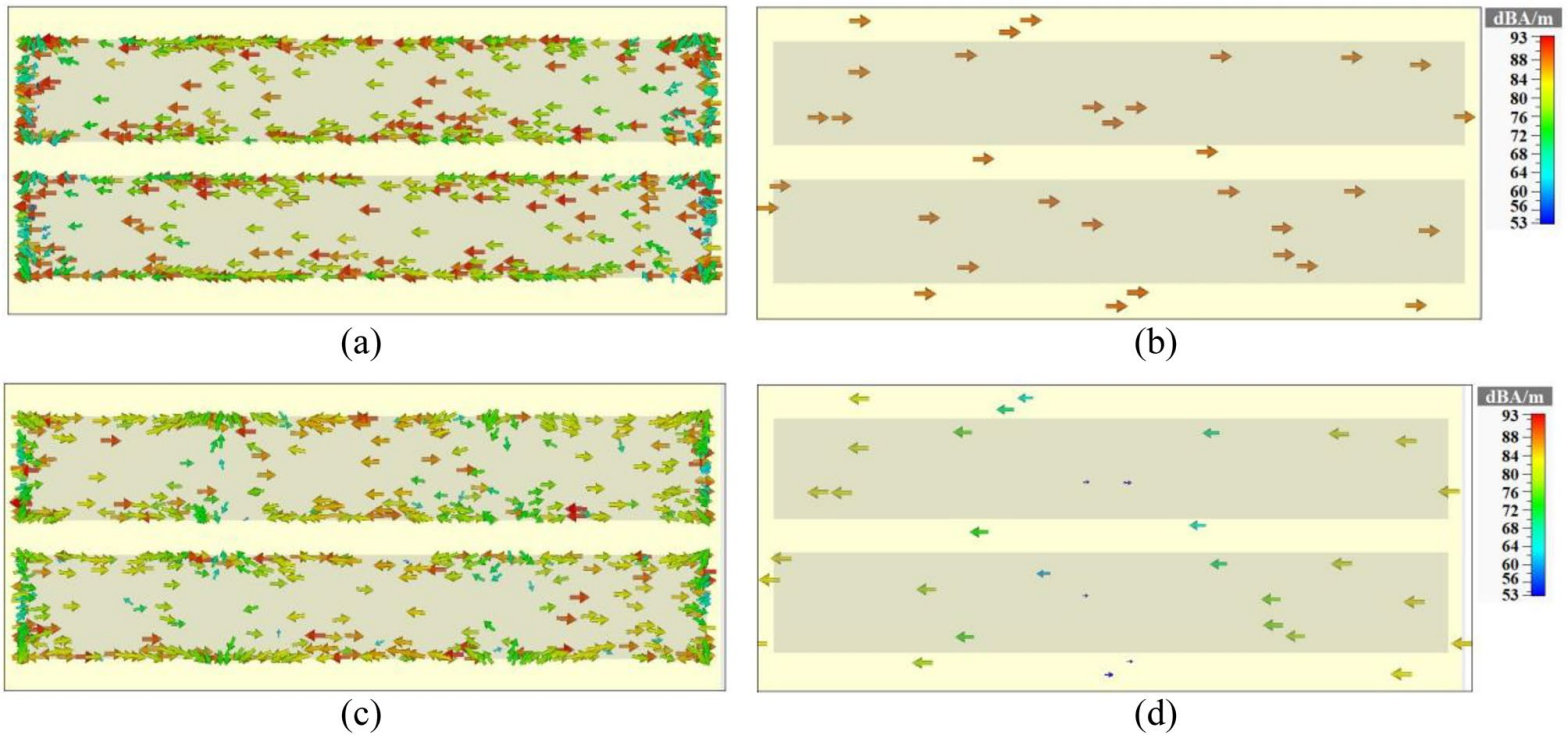
Surface current distributions of the metamaterial of Fig. 1 at 1.23 THz on (**a**) resonators surface and (**b**) gold reflector surface, at 4.21 THz on (**c**) resonators surface and (**d**) gold reflector surface.

The surface current distributions of the proposed multi-functional metamaterial of Fig. 1 at 1.23 THz for the resonator layer and the gold metal layer are obtained and given in Fig. 6a,b, respectively. The currents on the resonator layer are in the opposite direction of the currents on the gold layer. So, the currents create a closed loop and the resonance at 1.23 THz is magnetic. The surface current distributions of the metamaterial at 4.21 THz for the resonator layer and the gold layer are respectively given in Fig. 6c,d. The surface current distributions are not making a closed loop which means that it is an electric-type resonance.

**Figure 7:**
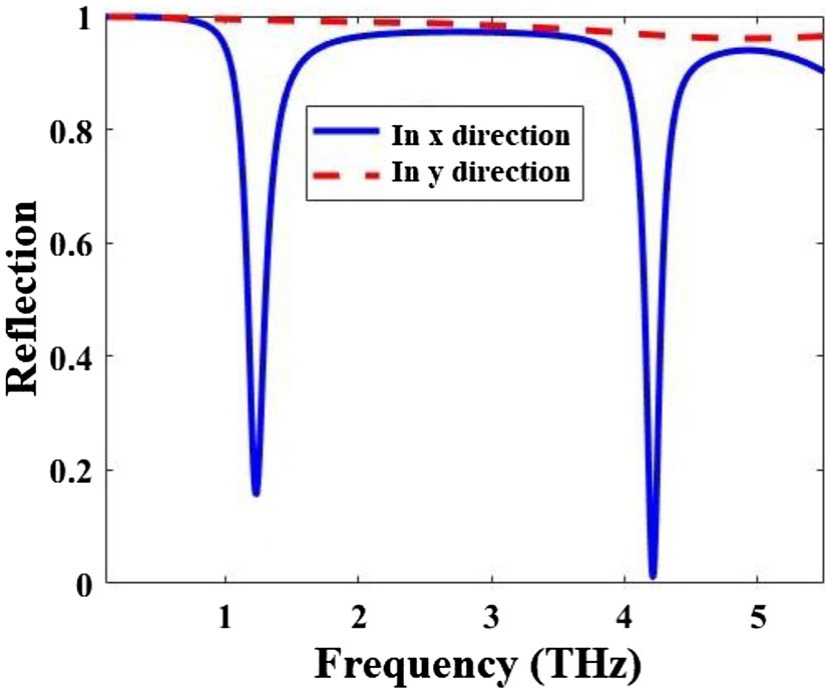
Reflection spectra of the multi-functional metamaterial of Fig. 1 in inverting (input = 0 *µ*_*c*_ = 0 and output = the spectrum in the y-direction, input = 1 *µ*_*c*_ = 1 and output = the spectrum in the x-direction), triple-band (the spectrum in the x-direction) and an ultra-broadband (the spectrum in the y-direction) mirrors performances.

The designed multi-functional metamaterial in Fig. 1 could act as an inverter. Inverter is a logic gate with one input and one output. We assume the *µ*_*c*_ of the graphene resonator layer as the input and the reflection value of the metamaterial as the output of the inverter. The reflection spectra of the metamaterial as an inverter are shown in Fig. 7. The truth table of the inverting performance of the metamaterial is given in Table 2.

**Table 2:** The truth table of the metamaterial in inverter performance for 1.23 and 4.21 THz.

Resonance frequency (THz)	Input state	*µ*_*c*_ (eV)	Reflection value	Output state
1.23	0	0	1	1
	1	1	0.15	0
4.21	0	0	0.97	1
	1	1	0.008	0

Moreover, the designed multi-functional metamaterial works as a triple-band mirror when the incident E field is in the x-direction. The metamaterial works as an ultra-broadband mirror when the incident E field is in the y-direction.

**Figure 8:**
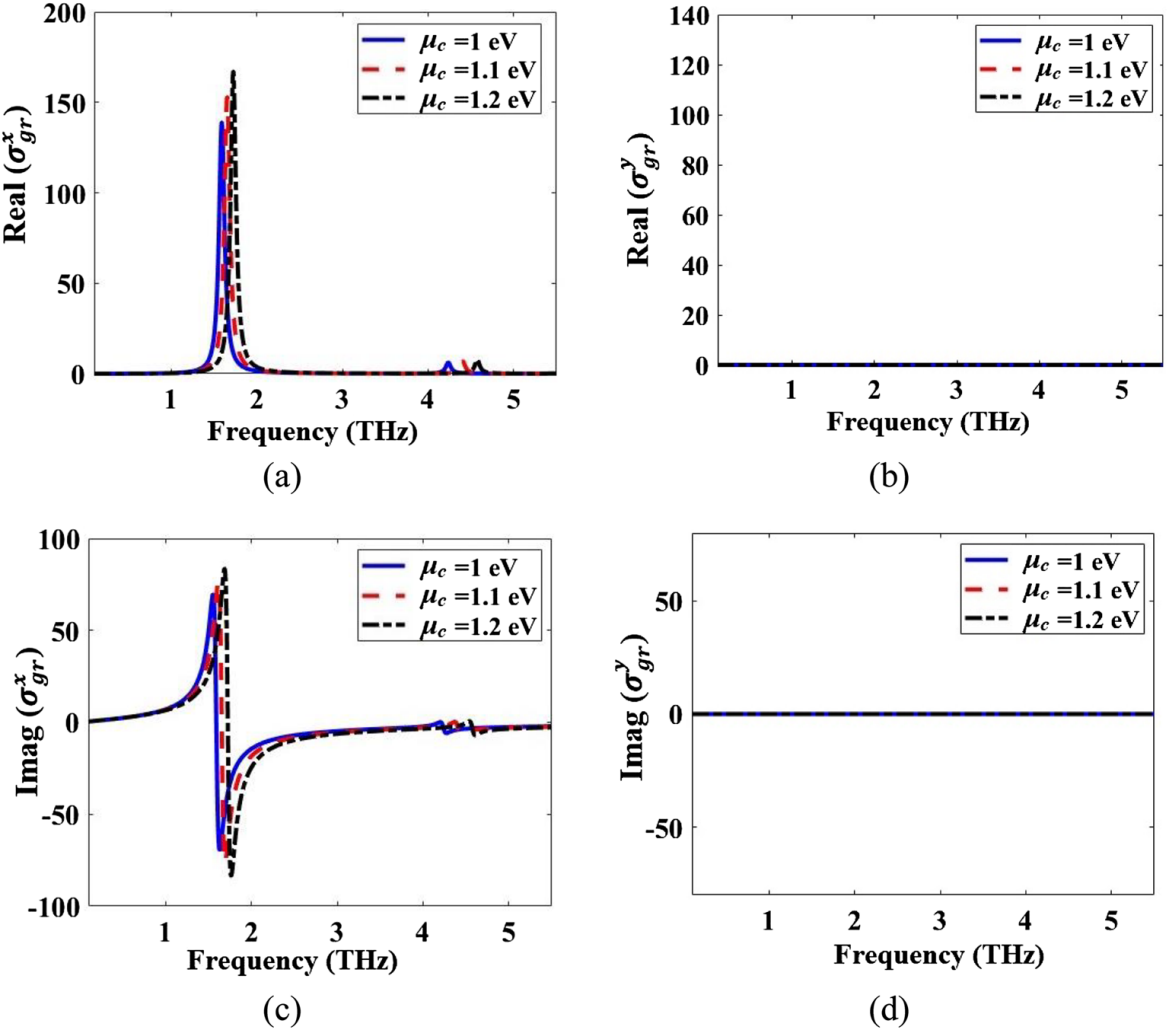
Real parts of the equivalent conductivities of (**a**) Eq. (7) and (**b**) Eq. (10). Imaginary parts of (**c**) Eq. (7) and (**d**) Eq. (10).

The structure containing the graphene resonator layer on the Quartz dielectric half-space (with a thickness of 500 µm) is simulated in CST and the reflection coefficients for this configuration are obtained. Then, the real and the imaginary parts of the equivalent conductivities in the x- and y-directions for the graphene resonator layer are obtained by Eqs. (7) and (10). The results are given in Fig. 8. The resonator layer is modeled as an impedance in the x-direction (Fig. 2a) so the real part of the conductivity in the x-direction is positive showing the resistive nature of the graphene resonator layer. The imaginary part of the conductivity in the x-direction has positive and negative parts showing the inductive and capacitive natures of the graphene resonator layer. The resonator layer is modeled as an OC in the y- direction (Fig. 2b) so the real and imaginary parts of the conductivity are zero in the y-direction.

**Figure 9:**
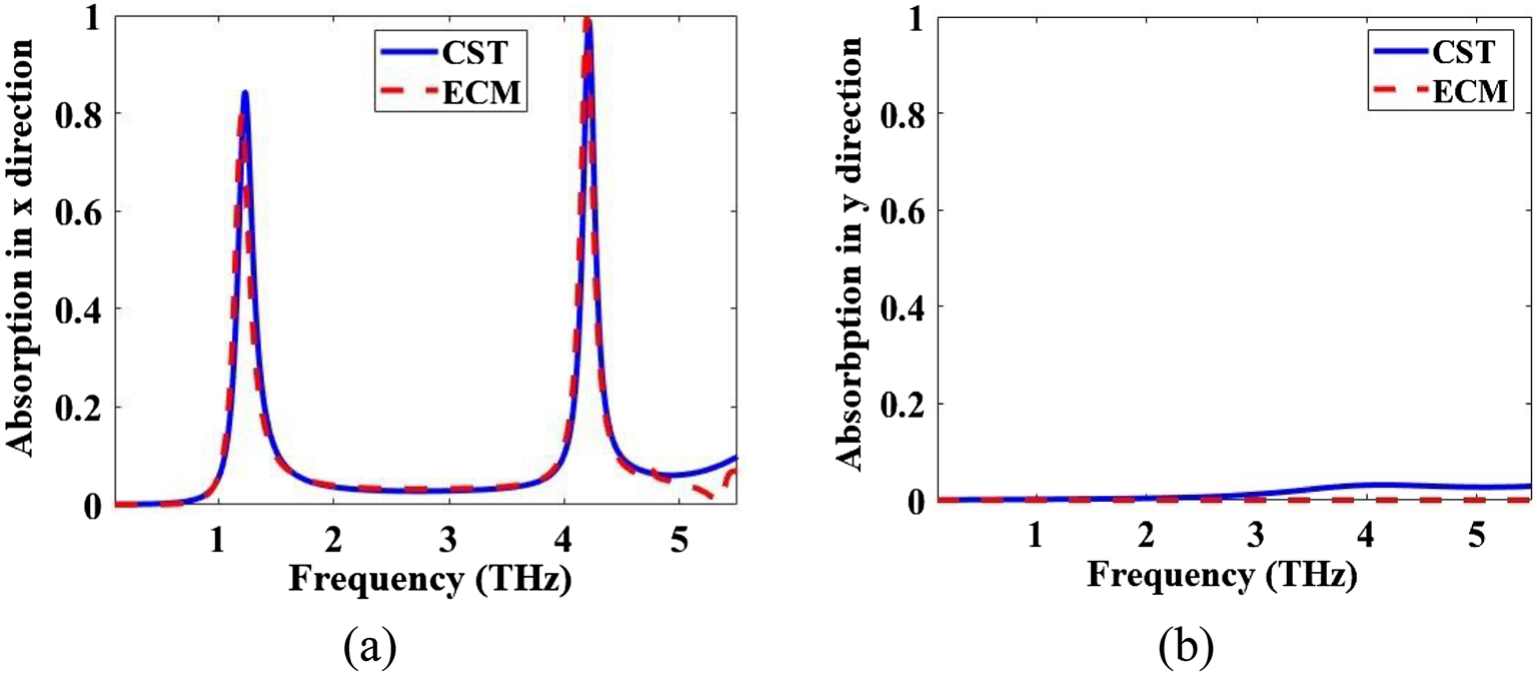
Comparison of CST and ECM absorption spectra of the metamaterial of Fig. 1 for the incident E field in the (**a**) x- and (**b**) y-directions.

**Figure 10:**
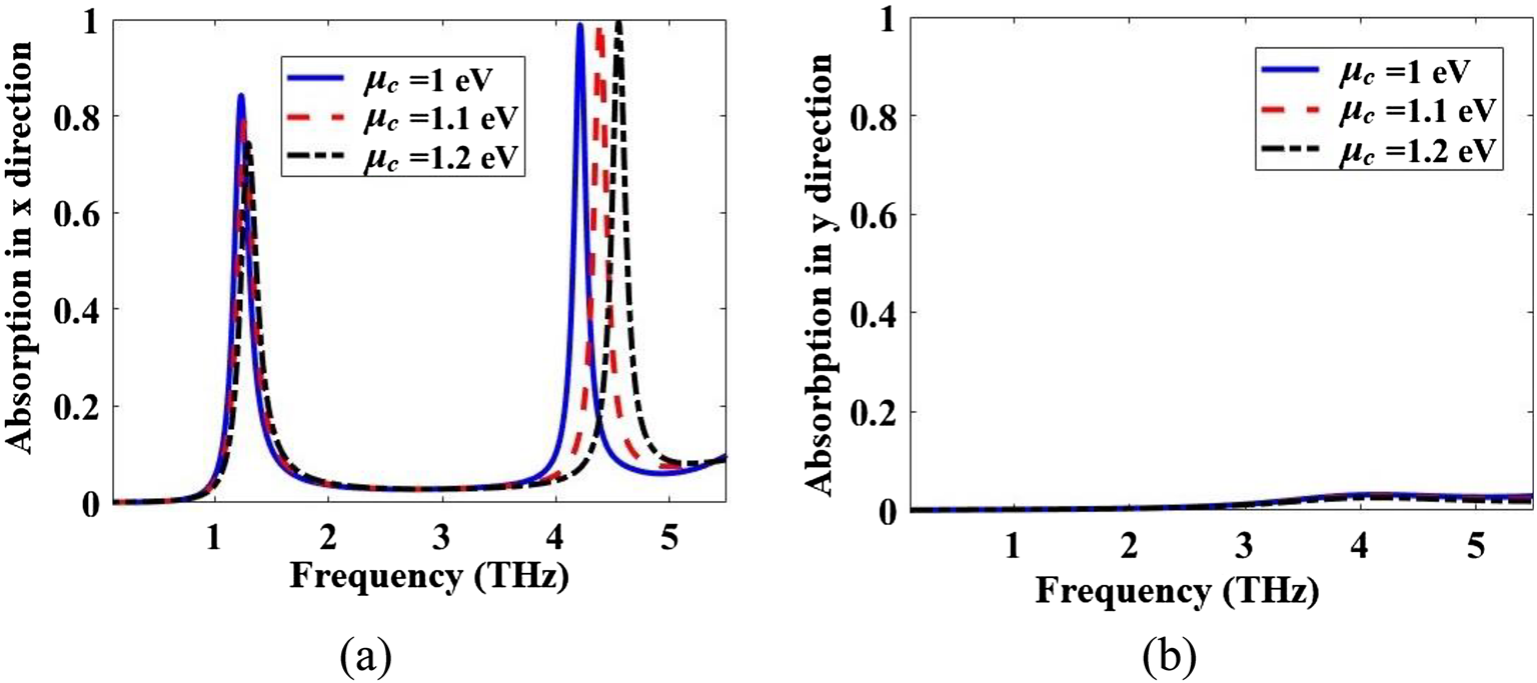
Absorption spectra of the metamaterial of Fig. 1 for three different *µ*_*c*_ for the incident E field in the (**a**) x- and (**b**) y-directions.

Absorption spectra of the multi-functional metamaterial in Fig. 1 are obtained both by CST and ECM methods for the incident E field in the x- and y-directions. The results with both methods are in good agreement and they are given in Fig. 9. To show the dynamical tunability of the absorption spectra of the designed metamaterial, it is simulated for three different *µ*_*c*_ and the results are given in Fig. 10. By increasing of the *µ*_*c*_, the resonance frequencies increase which exhibits a blueshift.

The designed multi-functional metamaterial is compared with previously published absorbers/mirrors including chiral absorbers/mirrors and anisotropic absorbers/mirrors in Table 3. The CD is defined as the absorption/transmission difference between right- and left-handed circular polarized waves (CD = CD_chi_ + CD_ani_) in the reported references of Table 3. The circular conversion dichroism (CCD) is defined as the transmission difference between left-to-right and right-to-left circular polarized conversion efficiencies^30^. The metamaterial is also compared with previously proposed switches in Table 4.

**Table 3:** Comparison of the absorbers/mirrors including chiral absorbers/mirrors and anisotropic absorbers/mirrors.

	Dynamically tunable	Frequency range	ECM	Max. absorption/reflection (%)	Max. LD/CD/CCD response/(%)	Multi-functional
^7^	Yes	0.5–4.5 THz	Yes	99 (absorption)	LD/94	Single-functional
^8^	Yes	0.3–4.5 THz	Yes	99 (reflection)	LD/96	Dual-functional
^10^	Yes	1–5.5 THz	Yes	100 (absorption)	LD/99	Single-functional
^11^	Yes	2–4.5 THz	No	100 (absorption)	CD/86.3	Dual-functional
^12^	Yes	0.5–1.5 THz	No	100 (absorption)	CD/80 LD/90	Dual-functional
^31^	No	7–10 GHz	No	93.2 (absorption)	CD/86	Single-functional
^32^	No	375–500 THz	No	80 (absorption)	CD/50	Single-functional
^33^	No	187.5–375 THz	No	50 (absorption)	CD/12	Single-functional
^34^	No	1.6–3.2 THz	No	97 (absorption)	CD/70	Single-functional
^35^	No	166.7–375 THz	No	not reported	CD/88 CCD/15	Single-functional
^36^	No	150–250 THz	No	85 (absorption)	CD/50	Single-functional
^37^	No	9.1–11 GHz	No	98 (reflection)	CD/93	Single-functional
^38^	No	300–375 THz	No	90 (reflection)	CCD/43	Single-functional
^39^	No	30–50 THz	No	99 (reflection)	CD/94	Single-functional
^40^	No	285–425 THz	No	90 (reflection)	CD/50	Single-functional
^41^	No	211–227 THz	No	80 (reflection)	CD/63	Single-functional
^42^	No	8–12 GHz	No	95 (reflection)	CD/88	Single-functional
This work	Yes	0.1–5.5 THz	Yes	100 (absorption)	LD/100	Quadruple-functional

**Table 4:** Comparison of the switches.

	Dynamically tunable	Frequency range (THz)	ECM	Max. extinction ratio (dB)
^43^	No	0.1–0.9	No	20.5
^44^	No	0.9–1.1	No	5.26
^45^	No	187–260	Yes	13.71
^46^	Yes	0.1–6.5	No	23
This work	Yes	0.1–5.5	Yes	33.01

The fabrication procedure of the designed metamaterial is not in the scope of this paper, but it can have the same procedure as explained in our previously published work^7^.

## Conclusion

In summary, we introduce and design a multi-functional anisotropic metamaterial containing two parallel graphene ribbons in each unit cell in the 0.1–5.5 terahertz (THz) region. The maximum absorption and linear dichroism of the metamaterial reached 100% and 100%, respectively. The metamaterial has a non-symmetric geometry, and it is polarization sensitive. The absorption/reflection spectrum of the metamaterial is obtained by use of the finite element method (FEM) in CST Software. The spectrum is dynamically tunable by the alternation of the applied bias voltage to graphene. Moreover, applications of the proposed metamaterial as a switch, an inverter, and a bi-functional mirror are studied. The maximum switching extinction ratio of the metamaterial reached 33.01 dB. It acts as a triple-band mirror for the incident electric field in the x-direction and an ultra-broadband mirror for the incident electric field in the y-direction. Using one device to reach four different functions (switching, inverting, triple-band mirror, and ultra-broadband mirror) can greatly reduce the size of the future THz systems saving material, time, and cost. An equivalent circuit modeling (ECM) approach by a simple MATLAB code has been presented to model the performance of the metamaterial. The FEM and ECM results are well-matched. The proposed metamaterial and its applications could be used in future THz devices and systems.

## Data Availability

The datasets used and/or analyzed during the current study are available from the corresponding author upon reasonable request.
